# Enhanced Soybean Productivity by Inoculation With Indigenous *Bradyrhizobium* Strains in Agroecological Conditions of Northeast Germany

**DOI:** 10.3389/fpls.2021.707080

**Published:** 2022-01-12

**Authors:** Richard Ansong Omari, Kun Yuan, Khoa Trinh Anh, Moritz Reckling, Mosab Halwani, Dilfuza Egamberdieva, Naoko Ohkama-Ohtsu, Sonoko D. Bellingrath-Kimura

**Affiliations:** ^1^Leibniz Centre for Agricultural Landscape Research (ZALF), Müncheberg, Germany; ^2^Faculty of Life Sciences, Humboldt-University of Berlin, Berlin, Germany; ^3^Institute of Global Innovation Research (GIR), Tokyo University of Agriculture and Technology, Tokyo, Japan; ^4^Institute of Agriculture, Tokyo University of Agriculture and Technology, Tokyo, Japan; ^5^Faculty of Biology, National University of Uzbekistan, Tashkent, Uzbekistan

**Keywords:** *Bradyrhizobium strains*, soybean productivity, biological N_2_ fixation, Central Europe, nodulation

## Abstract

Commercial inoculants are often used to inoculate field-grown soybean in Europe. However, nodulation efficiencies in these areas are often low. To enhance biological nitrogen (N) fixation and increase domestic legume production, indigenous strains that are adapted to local conditions could be used to develop more effective inoculants. The objective of this study was to assess the ability of locally isolated *Bradyrhizobium* strains to enhance soybean productivity in different growing conditions of Northeast Germany. Three indigenous *Bradyrhizobium* isolates (GMF14, GMM36, and GEM96) were tested in combination with different soybean cultivars of different maturity groups and quality characteristics in one field trial and two greenhouse studies. The results showed a highly significant strain × cultivar interactions on nodulation response. Independent of the *Bradyrhizobium* strain, inoculated plants in the greenhouse showed higher nodulation, which corresponded with an increased N uptake than that in field conditions. There were significantly higher nodule numbers and nodule dry weights following GMF14 and GMM36 inoculation in well-watered soil, but only minor differences under drought conditions. Inoculation of the soybean cultivar Merlin with the strain GEM96 enhanced nodulation but did not correspond to an increased grain yield under field conditions. USDA110 was consistent in improving the grain yield of soybean cultivars Sultana and Siroca. On the other hand, GMM36 inoculation to Sultana and GEM96 inoculation to Siroca resulted in similar yields. Our results demonstrate that inoculation of locally adapted soybean cultivars with the indigenous isolates improves nodulation and yield attributes. Thus, to attain optimal symbiotic performance, the strains need to be matched with specific cultivars.

## Introduction

Soybean [*Glycine max* (L.) Merr.] consumption as food and feed for livestock in Europe has increased significantly in recent decades, although domestic production has increased marginally. The European Union encourages domestic grain legume production to reduce reliance on imports, to minimize the negative environmental impacts associated with intensive cereal production (Watson et al., 2017), and to diversify cropping systems (Hufnagel et al., 2020). Meanwhile, most farmers in Europe prefer to invest in cereal crops due to the high economic returns and lower risk of losses compared with grain legumes (Voisin et al., 2013), although Reckling et al. (2018) found that their yield stability is similar to that of other spring crops. Soybean cultivation in Central Europe is still relatively new (Zimmer et al., 2016), despite an almost twofold increase in production area between 2013 and 2018 (Krautgartner et al., 2018). In Germany, while domestic soybean production is low, cultivation is mainly concentrated in the south with a small and almost non-existent contribution from the northern part. However, there is great potential to expand soybean production areas in some cool climates and non-traditional growing areas of Europe due to increasing temperatures (de Visser et al., 2014; Feng et al., 2021). A study by Karges et al. (2022) also highlighted the agro-economic prospects for expanding soybean production beyond its current northerly limit in Europe.

Biological N_2_ fixation (BNF) ensures the utilization of atmospheric nitrogen (N), which translates into improved soil fertility, increased crop productivity, and an economically sustainable production system (Choudhury and Kennedy, 2004; Zander et al., 2016). Efficient BNF depends on proficient symbiotic interactions between the legume host and rhizobium in a range of environmental conditions that favor the activities of participating organisms. This interaction has implications for the stability of the performance of legume inoculants (Gunnabo et al., 2019) and thus reflects stable and increased legume production. Environmental stress factors such as temperature, drought, salinity, pH, biotic competition, etc. affect both the host and rhizobium response, leading to a reduction in BNF efficiency (Laranjo and Oliveira, 2011; Goyal et al., 2021). According to Cernay et al. (2015), the high susceptibility of BNF to environmental stress factors is one of the major drivers of the high grain legume yield variability in Europe. Therefore, optimizing legume-rhizobium interactions by targeting inoculant strains to specific soybean cultivars presents a promising option to increase the efficiency of N symbiosis and crop productivity in Central Europe.

Although commercial rhizobia strains have in the past been used to inoculate field-grown soybean plants in Germany, nodulation efficiency has often been low compared with the origin where the strains were isolated (Kadiata et al., 2012; Schmidt et al., 2015). To enhance BNF and increase domestic legume production, native or indigenous rhizobium strains that are adapted to local conditions could be targeted for the development of inoculants. Genetically stable rhizobium strains to harsh soil conditions may enable higher BNF and increase grain yield in soybean compared with commercial inoculants (Hungria and Vargas, 2000; Rahmani et al., 2008). Yuan et al. (2020) successfully isolated promising rhizobium strains from arable fields in northern Germany. Some of the isolates in their pot experiment were tolerant to cold conditions and showed great potential to enhance soybean growth under low-temperature conditions. However, the effectiveness of these strains on soybean productivity has not yet been tested under field conditions in Germany.

Thus, for the selection of effective strains for future use as commercial inoculants to improve soybean production, there is a need to test the efficacy of the isolated strains of Yuan et al. (2020) under growing conditions in Central Europe. The goal of this study was to assess the ability of indigenous *Bradyrhizobium* strain(s) to enhance soybean productivity under varying growing conditions in Northeast Germany in greenhouse and field studies.

## Materials and Methods

### Description of the Study Site

Two greenhouse experiments and one field trial were conducted at the experimental station at the Leibniz Center for Agricultural Landscape Research, Müncheberg, Germany (52°31′ N; 14°7′ E; 60 m). Soils for both greenhouse and field studies were from sites with no previous soybean cropping history. The soil in the area is classified as Podzoluvisol to Arenosol and is located 62 m above sea level. The area is characterized by droughts in spring and summer. The mean annual precipitation is 533 mm, and the mean long-term annual temperature is 8.5°C.

### Trial Description

#### Greenhouse Experiment 1

This greenhouse experiment was an initial screening intended to study the interaction effects of representative soybean cultivars of Central Europe and the different *Bradyrhizobium* isolates on nodulation and biomass development. Five soybean cultivars of different maturity groups, growth and quality characteristics, namely, Comandor (000), Merlin (000), Shouna (000), Sultana (000), and Siroca (00) (Table 1), were tested in combination with three indigenous *Bradyrhizobium* isolates (GMF14, GMM36, and GEM96) and compared with a non-inoculated control (Table 2). These are among the commonly cultivated cultivars in Central Europe. *Bradyrhizobium* isolates were previously isolated from arable soils in Northeast Germany (Fehrow and Müncheberg). A 4 × 5 factorial combination involving *Bradyrhizobium* isolates and soybean cultivars, respectively, in a complete randomized design was set for the study. Each treatment was replicated 3 times.

**TABLE 1:** Characteristics of soybean cultivars.

Cultivar	Date of registration	Maturity group	Maturity	Height	Growth type	Thousand-grain weight	Protein content	Oil content
Comandor	2016	000	5	5	Semi-determinate	4	5	5
Merlin	1997	000	2	3	Indeterminate	1	5	7
Shouna	2015	000	4	5	Indeterminate	3	6	5
Sultana	2009	000	3	3	Semi-determinate	4	7	5
Siroca	2017	00	5	4	Semi-determinate	5	7	4

*German descriptive variety list (2021) for cv. Comandor and Austrian descriptive variety list (2021) for all other cultivars and growth type from the variety list of the German soybean association (2021).*

**TABLE 2:** Characteristics of *Bradyrhizobium* strains.

Strain	Host cultivar	Sampling site	Classification (MSLA)	Growth	Temperature (°C)	pH	NaCl (%)
GMF14	Merlin	Müncheberg	*Bradyrhizobium* sp.	+	4–37	4.5–10	4%
GMM36	Merlin	Müncheberg	*Bradyrhizobium* sp.	+	15–37	4.5–10	0%
GEM96	Enrei	Fehrow	*Bradyrhizobium* sp.	+	15–37	4.5–10	0%

*Yuan et al. (2020).*

Each *Bradyrhizobium* isolate was cultured in yeast extract mannitol (YEM) liquid medium for 5 days at 28°C. The cells were then concentrated and washed twice with sterile distilled water. Each bacterial suspension was adjusted to a concentration of 10^8^ cells ml^–1^.

Seeds of each cultivar were first surface-sterilized with 70% alcohol for 1 min, followed by 3% sodium hypochlorite for 2 min. The seeds were rinsed 8 times with sterile distilled water to remove traces of alcohol and sodium hypochlorite. Seeds were then dried in sterile towel paper.

Each surface sterilized seed was inoculated by thoroughly mixing 65 ml liquid culture with 1 kg seeds and allowing them to dry at room temperature for 2 h before sowing. Sowing was carried out on 10th September 2019. Three healthy seeds of uniform size were sown in each box (10 cm × 8 cm × 8 cm) containing 1 kg soil and were later thinned to one plant per box of comparable height and vigor after 2 weeks. Soybean plants were grown for 40 days (V5 growth stage) at an average temperature of 22°C under a 16-h light and 8-h dark photoperiod as employed by Yuan et al. (2020). The average relative humidity during the study was 50%. Pots were watered to field capacity with distilled water at 4-day intervals until the termination of the experiment. No chemical fertilizers were applied during the growth of soybean plants. The physicochemical properties of the soil are presented in Table 3.

**TABLE 3:** Selected physicochemical properties of the soil used.

Variable (Unit)	Concentration
Total carbon (g kg^–1^)	6.5 ± 0.71
Total nitrogen (g kg^–1^)	0.5 ± 0.03
Total sulfur (g kg^–1^)	0.2 ± 0.02
Available phosphorus (mg 100 g^–1^)	20.9 ± 0.35
Potassium (mg 100 g^–1^)	12.1 ± 0.87
Magnesium (mg 100 g^–1^)	1.4 ± 0.08
pH (H_2_O)	6.7
Sand (%)	61
Silt (%)	27
Clay (%)	12

*±, Standard deviation of means.*

#### Greenhouse Experiment 2

This study was conducted to evaluate the extent to which the symbiotic performance of the *Bradyrhizobium* strains is impacted by varying soil moisture levels. Sultana, one of the cultivars that showed a moderate response to inoculation in greenhouse Experiment 1 in terms of nodulation and shoot biomass growth was used. The three *Bradyrhizobium* strains used in greenhouse Experiment 1 plus a reference strain (*Bradyrhizobium diazoefficiens* USDA110) were inoculated to Sultana under two moisture levels. Non-inoculated soybean seeds were used as a control.

Seed sterilization and inoculum preparation were performed as explained in greenhouse Experiment 1. In this study, inoculation was performed by mixing 10 g autoclaved peat (141°C for 24 min) with 65 ml liquid culture to form a slurry. Peat was added to reduce the excessive softening of the seed coat, which affected germination in greenhouse Experiment 1. Then, 1 kg soybean seeds were thoroughly mixed with the slurry until each seed was sufficiently coated. Sowing was carried out on 13th June 2020 as explained in greenhouse Experiment 1. The pots were arranged in a complete randomized design and each treatment was replicated four times.

Three days after sowing, two moisture levels namely well-watered conditions (irrigation at 80% soil moisture) and drought conditions (irrigation at 40% soil moisture) were imposed on the respective treatment until termination of the study (50 days after sowing). Irrigation drippers were installed in each pot, and moisture was supplied when necessary. The well-watered treatment received 70 ml of water 4–5 times a week. The drought treatment received 35 ml of water 1–2 times a week. Soil moisture conditions were monitored with a UMP-1 BT soil moisture sensor (Umwelt-Geräte-Technik GmbH, Müncheberg, Germany) (Egamberdieva et al., 2019). Similar growth conditions as greenhouse Experiment 1 were set for this study.

#### Field Trial

This study was intended to evaluate different soybean cultivar responses to *Bradyrhizobium* strain inoculation under field conditions. Three soybean cultivars adapted to Northeast Germany from the list of cultivars used in greenhouse Experiment 1 were selected for this study. They were tested in combination with four *Bradyrhizobium* strains (GMF14, GMM36, GEM96, and USDA110 (same strains used in greenhouse Experiment 2) and a non-inoculated control. The treatments were laid out in a randomized complete block design with 5 × 3 factorial combinations of *Bradyrhizobium* and soybean cultivars, respectively, and each treatment was replicated six times. The trial was conducted in the 2020 cropping season (May–October) at the site where soil for both greenhouse experiments was collected.

Soybean seed sterilization, inoculum preparation, formulation, and inoculation were performed as explained in greenhouse Experiment 2. Each plot size measured 11.5 m × 1.8 m, and alleys of 3.5 and 0. 5 m were left between blocks and plots, respectively. Soybean seeds were sown at 80 seeds m^–2^ at a spacing of 37.5 cm between rows on 5th May 2020. To avoid cross-contamination, the plot seeder was thoroughly cleaned after each treatment with a different inoculant by following a modified approach of Zimmer et al. (2016). First, 3 kg of barley seeds were treated with 70% alcohol and immediately run through the seeder, followed by a thorough cleaning with pneumatic pressure. Blanket phosphorus, potassium, magnesium, and sulfur fertilizer (P/K/Mg/S 9:42:2:10) and kieserit (Mg/S 30:40) were applied to the field at 200 kg ha^–1^ before sowing.

### Measurements

In both greenhouse studies, plant shoots were cut with scissors at 1 cm above the soil surface. Then, the soil in each pot was emptied on a white sheet covered with transparent plastic material. The plant was carefully removed to obtain unharmed roots and nodules. All adhering soil particles were rinsed off with a stream of water, and nodules were gently detached from the roots. The number of nodules on the roots was manually counted. All fresh nodules and shoot samples were dried in an oven at 60°C for 48 h. The dry weights of all samples were determined with a weighing balance. Dry shoot samples were later milled for the determination of shoot N uptake.

In the field trial, aboveground biomass samples were collected from two inner rows of each plot 0.5 m in length at the R3 growth stage. Five plants from the inner rows of each plot were randomly selected, and their roots together with nodules were gently excavated with a spade. The shoot dry weight, nodule number, nodule dry weight, and shoot N uptake were determined as was conducted in both greenhouse studies. At physiological maturity, all soybean plants were harvested with a plot combine harvester. Grain yield (kg ha^–1^) and thousand grain weight (TGW) were determined at 86% dry matter. Shoot N analysis was performed by the dry combustion method using a CNS elemental analyzer (Leco Instruments GmbH). The crude protein and oil contents in the grains were measured with near-infrared reflectance spectroscopy (NIRS) (Inframatic 9500, Perten Instruments, Sweden).

Soil sampling was performed before sowing at 0–15 cm depth from up to 12 random points. They were then passed through a 2 mm mesh and air-dried before further analyses. Total soil carbon, N, and sulfur were determined by dry combustion using a CNS elemental analyzer. Phosphorus, potassium, and magnesium contents were analyzed by ICP-OES (iCAP 6300 DUO, Thermo Fisher Scientific GmbH) via the Mehlich-3 extraction method (Sims, 2009). Soil pH was measured in a 1:2 (v/v) soil-water suspension with a Robotic Titrosampler (Deutsche Metrohm GmbH & Co., KG). The particle size distribution was determined by wet sieving and sedimentation with the Köhn-Pipette method after digesting 10 g soil with 100 ml H_2_O_2_ and chemical dispersion using Na_4_P_2_O_7_ (Hartge and Horn, 1992).

### Data Analysis

Analysis of variance (ANOVA) was performed using the generalized linear model procedure (GLM) of the software package SAS 9.2 (SAS Institute 2008) after checking for normality and homogeneity of variances. In the field study, nodulation parameters, shoot dry weight, N uptake, TGW, pod number, grain weight, protein content, and oil content were analyzed using the two-way ANOVA model below:


$$Y_{ikl}=\mu +\alpha_{i}+\gamma_{k}+\alpha \gamma_{ik}+\beta_{l}+\delta_{l}+e_{ikl}$$

where,

*Y*_*ikl*_ = responses (Yield, …)

μ = intercept (overall mean response of all observations)

*a_i_* = the soybean cultivar effect (fixed effect) of the *i^th^* level of α

γ_*k*_ = the *Bradyrhizobium* strain effect (fixed effect) of the *k^th^* level of γ

αγ_*ik*_ = interaction effect of soybean cultivar by *Bradyrhizobium* strains of the *i^th^* level of α with the *k^th^* level of γ

β_*l*_ = the block effect (random effect) of the *l^th^* level of β

δ_*l*_ = the restriction error associated with the *l^th^* block

*e*_*ikl*_ = the random error

The same model was used to analyze the responses in greenhouse Experiments 1 and 2. However, the blocking effect was not considered in analyzing the responses in either greenhouse experiment. In greenhouse Experiment 1, soybean cultivar and *Bradyrhizobium* strain were considered fixed factors, whereas, in greenhouse Experiment 2, *Bradyrhizobium* strain and moisture regimes were modeled as fixed factors. All pairwise comparisons were performed using the Tukey-test (*p* ≤ 0.05). The effects of all factors and their interactions were assessed using the standard error of difference (mean SED).

## Results

### Shoot Biomass Yield

**FIGURE 1:**
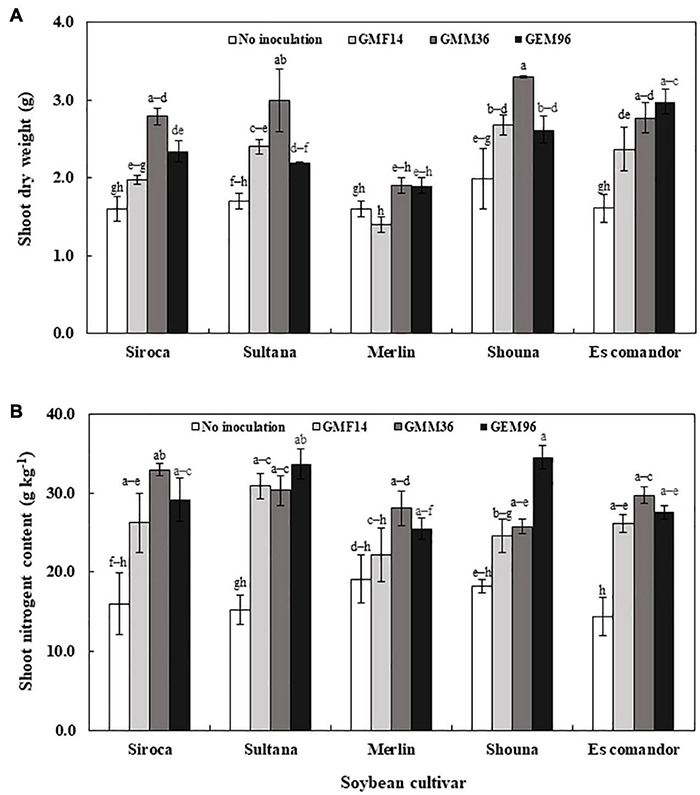
Shoot dry weight **(A)** and shoot nitrogen content **(B)** following inoculation of different Bradyrhizobium strains to five soybean cultivars in the first greenhouse experiment. The error bars are standard deviation of three replications (*n* = 3). Values followed by the same letters on each bar are not significantly different at *p* ≤ 0.05.

**FIGURE 2:**
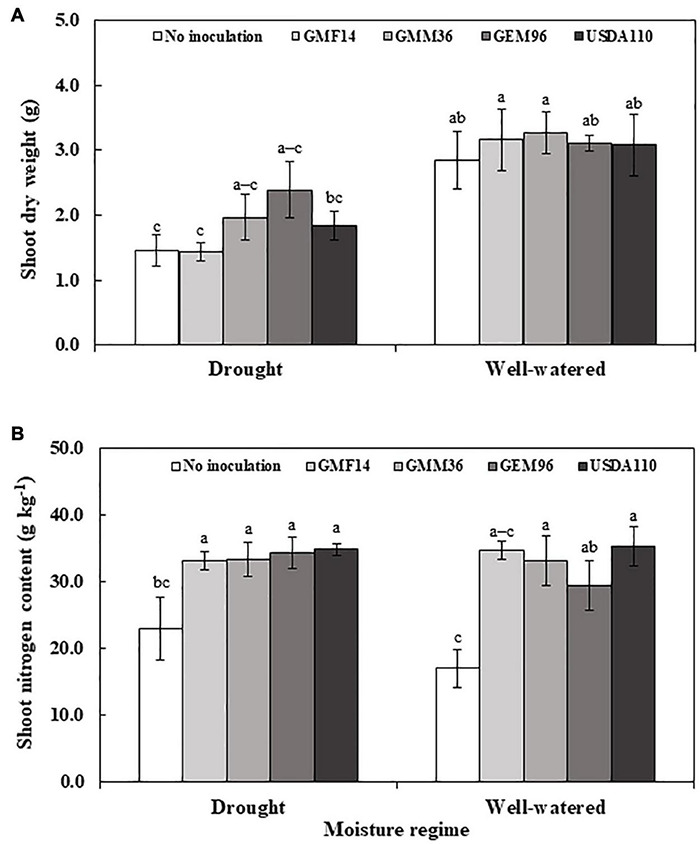
Shoot dry weight **(A)** and shoot nitrogen content **(B)** of soybean plant (Cv. sultana) inoculated with different Bradyrhizobium strains in two moisture regimes in the second greenhouse experiment. The error bars are standard deviation of four replications (*n* = 4). Values followed by the same letters on each bar are not significantly different at *p* ≤ 0.05.

**FIGURE 3:**
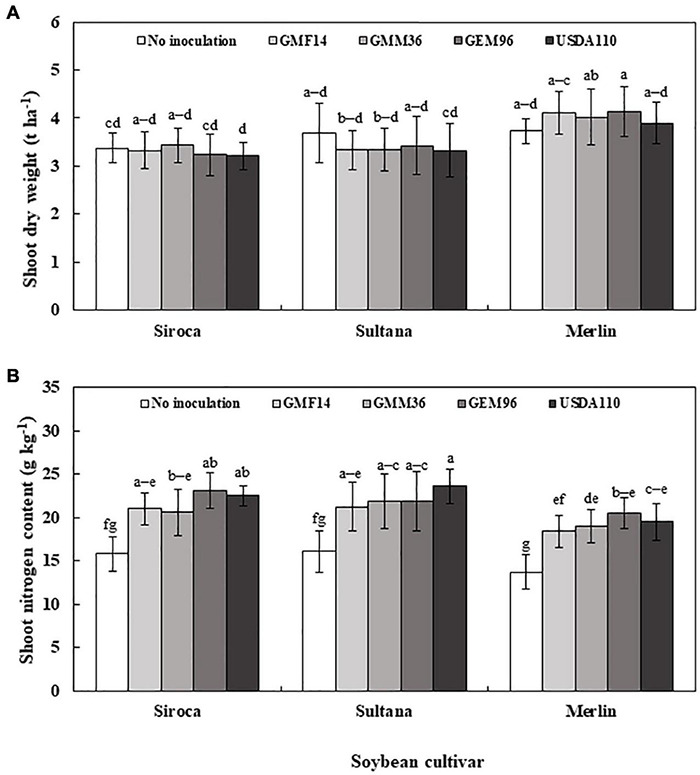
Shoot dry weight **(A)** and shoot nitrogen content **(B)** of three soybean cultivars inoculated with different Bradyrhizobium strains in field conditions. The error bars are standard deviation of six replications (*n* = 6). Values followed by the same letters on each bar are not significantly different at *p* ≤ 0.05.

In greenhouse experiment 1, shoot dry weight was significantly influenced by soybean cultivar, *Bradyrhizobium*, and soybean cultivar *× Bradyrhizobium* interactions (Table 4). Across the five soybean cultivars, the shoot dry weight in cv. Shouna was on average higher than that in the other cultivars irrespective of *Bradyrhizobium* inoculation (Figure 1A). GMM36 inoculation consistently induced the highest significant shoot dry biomass. In greenhouse Experiment 2, shoot dry weight was significantly affected by moisture regime but not by *Bradyrhizobium* inoculation (Table 4). No significant interaction was detected. The shoot dry weight under well-watered conditions was 70% higher compared to the drought conditions (Figure 2A and Supplementary Table 3). Under field conditions, soybean cultivar significantly affected shoot dry weight, but *Bradyrhizobium* inoculation did not influence shoot dry weight. No significant soybean variety *× Bradyrhizobium* interaction was detected. Independent of inoculation, Merlin showed the highest significant shoot dry weight of 3.92 t ha^–1^ (Figure 3A and Supplementary Table 4).

**TABLE 4:** Summary of ANOVA for the influence of cultivar, bradyrhizobial type, moisture and their interrelations on nodulation and yield in the two greenhouse experiments and field trial.

Source	*p*-value									
	df	Nodule no.	Nodule dry wt. (g)	Biomass wt. (g)	Shoot N (g kg^–1^)	TGW (g)	Pod no.	Protein content (g kg^–1^)	Oil content (g kg^–1^)	Grain yield (kg ha^–1^)
**Greenhouse exp. 1**										
Cultivar (C)	4	<0.001	<0.001	<0.001	0.04	ND	ND	ND	ND	ND
Bradyrhizobia (B)	3	<0.001	<0.001	<0.001	<0.001	ND	ND	ND	ND	ND
C × B	12	<0.001	<0.001	<0.001	0.006	ND	ND	ND	ND	ND
**Greenhouse exp. 2**										
Bradyrhizobia (B)	4	<0.001	<0.001	0.20	<0.001	ND	ND	ND	ND	ND
Moisture (M)	1	<0.001	<0.001	<0.001	0.14	ND	ND	ND	ND	ND
B × M	4	<0.001	<0.008	0.46	0.21	ND	ND	ND	ND	ND
**Field trial**										
Cultivar (C)	2	0.51	<0.001	<0.001	<0.001	<0.001	0.02	<0.001	<0.001	<0.001
Bradyrhizobia (B)	4	<0.001	<0.001	0.23	<0.001	<0.001	0.009	<0.001	<0.001	<0.001
C × B	8	0.07	<0.001	0.46	0.40	<0.001	0.53	0.08	0.80	<0.001

*p-value represents significant effects at the 5% level. ANOVA, Analysis of variance; ND, Not determined.*

### Shoot N Uptake

Shoot N content was significantly influenced by cultivar, *Bradyrhizobium*, and soybean cultivar *× Bradyrhizobium* interaction in greenhouse Experiment 1 (Table 4). Inoculation with GMM36 and GEM96 resulted in consistently higher shoot N contents (Figure 1B). GMM36 and GEM96 inoculation significantly increased the shoot N content by an average of 79% compared with the non-inoculated control. On average, cv. Sultana (27.6 g kg^–1^) and Siroca (26.1 g kg^–1^) had the highest shoot N content, while the lowest was in Merlin (23.7 g kg^–1^) irrespective of *Bradyrhizobium* inoculation.

In greenhouse Experiment 2, the shoot N content was significantly influenced by *Bradyrhizobium* inoculation but not by the moisture regime (Table 4). No significant interaction was detected. Inoculation increased the shoot N content by an average of 68% over non-inoculated control (Figure 2B and Supplementary Table 3). Under field conditions, the shoot N content was significantly influenced by the soybean cultivar and *Bradyrhizobium* inoculation (Table 4). Across the tested *Bradyrhizobium* strains and independent of soybean cultivar, inoculation with USDA110 and GEM96 induced the highest significant shoot N, 21.91 and 21.89 g kg^–1^, respectively (Figure 3B). Shoot nitrogen increased by an average of 39% between the inoculated and non-inoculated control under field conditions. Consistent with greenhouse Experiment 1, Sultana and Siroca significantly had the highest shoot N uptake (Supplementary Table 4).

### Nodulation

**FIGURE 4:**
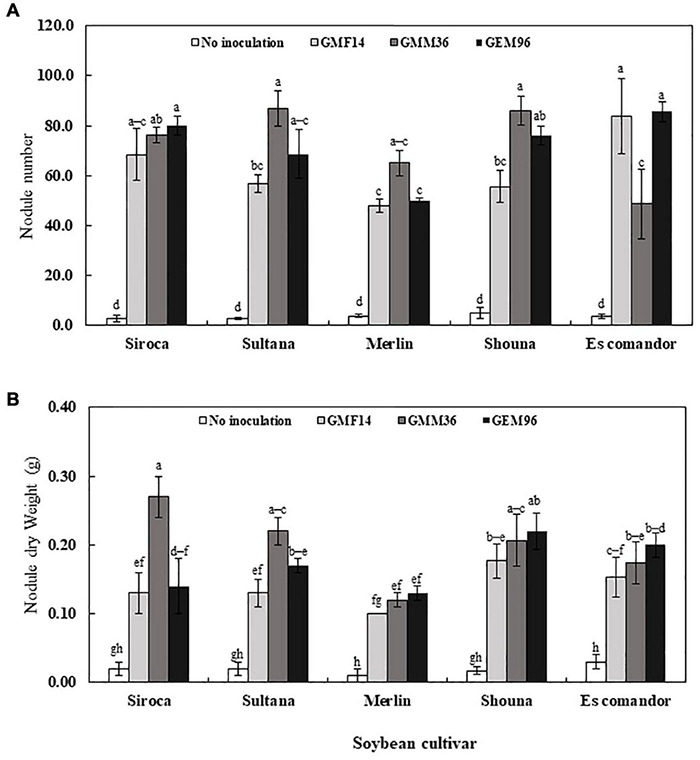
Soybean nodule number **(A)** and nodule dry weight **(B)** following inoculation of different Bradyrhizobium strains to five soybean culuVars in the first greenhouse experiment. The error bars are standard deviation of three replications (*n* = 3). Values followed by the same letters on each bar are not significantly different at *p* ≤ 0.05.

Nodule number and nodule dry weights were significantly affected by cultivar, *Bradyrhizobium* strain, and soybean cultivar *× Bradyrhizobium* interaction in greenhouse Experiment 1 (Table 4). Consistently, low nodule number and nodule dry weights were observed when strains were inoculated to Merlin. Irrespective of soybean cultivar, inoculation with strains GMM36 and GEM96 induced a significantly higher average nodule number, 72 plant^–1^ (Figure 4A). GMM36 inoculation resulted in the highest average nodule dry weight, 0.20 g (Figure 4B). A similar average number of nodules was observed in Siroca, Sultana, Shouna, and Comandor but was significantly higher than that in the Merlin group.

**FIGURE 5:**
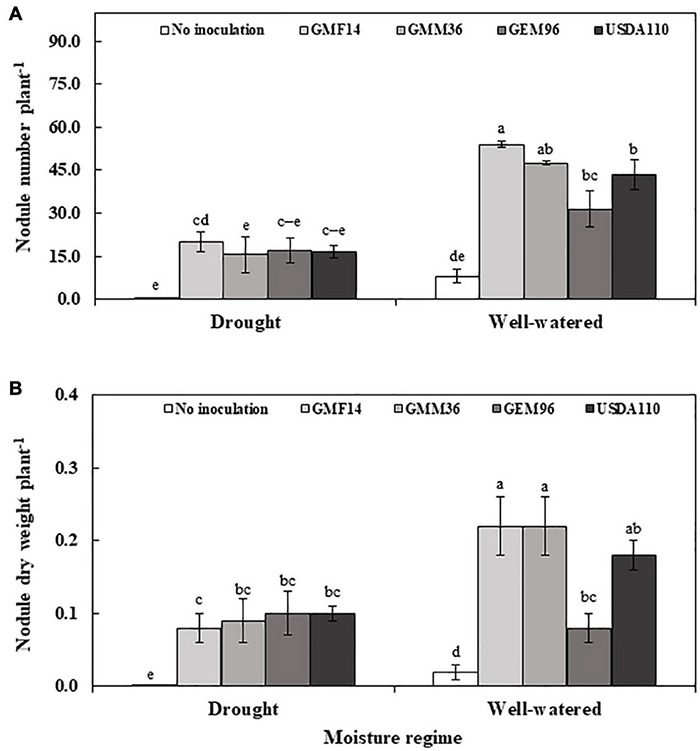
Nodule number **(A)** and nodule dry weight **(B)** of soybean plant inoculated with different Bradyrhizobium strains in two moisture regimes in the second greenhouse experiment. The error bars are standard deviation of four replications (*n* = 4). Values followed by the same letters on each bar are not significantly different at *P* ≤ 0.05.

In greenhouse Experiment 2, *Bradyrhizobium* strain, moisture regime, and their interaction significantly influenced nodulation (Table 4). Consistently, a higher significant nodule number and nodule dry weights were observed in GMF14 and GMM36 inoculation under well-watered moisture conditions (Figure 5). However, under drought conditions, minimal differences in nodulation were observed among the *Bradyrhizobium* strains. There were 2–3 times higher nodule numbers and nodule dry weights in the well-watered conditions than in the drought conditions.

**FIGURE 6:**
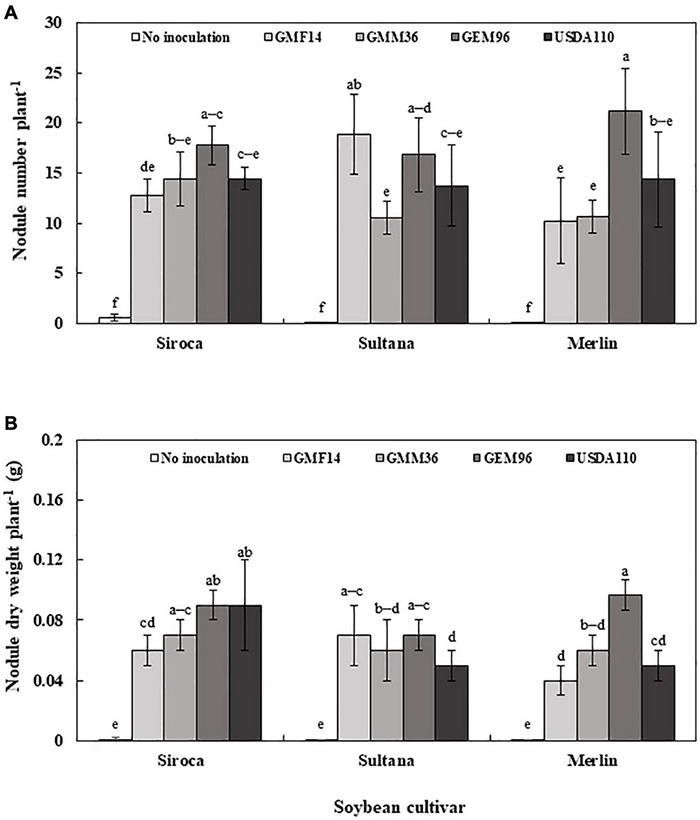
Nodule number **(A)** and Nodule dry weight **(B)** of three soybean culuVars inoculated with different Bradyrhizobium strains in field conditions. The error bars are standard deviation of six replications (*n* = 6). Values followed by the same letters on each bar are not significantly different at *p* ≤ 0.05.

In the field experiment, *Bradyrhizobium* inoculation, soybean cultivar, and their interaction significantly affected nodule dry weight (Table 4). However, only the *Bradyrhizobium* strain significantly influenced the number of nodules formed. Regardless of soybean cultivar, inoculation with GEM96 resulted in a significant increase in nodule number (18 plant^–1^) and nodule dry weight (0.08 g plant^–1^) (Supplementary Table 4). The highest nodule number (21 plant^–1^) and nodule dry weight (0.09 g plant ^–1^) were observed when Merlin was inoculated with GEM96 (Figure 6). In contrast to greenhouse Experiment 1, a similar number of nodules were observed among the three cultivars, although there were significantly heavier nodules on Siroca than on Sultana and Merlin.

### Pod Number and Grain Weight

Pod number and TGW were significantly influenced by soybean cultivar and by *Bradyrhizobium* strain (Table 4). While the soybean cultivar × *Bradyrhizobium* strain interaction was significant for TGW, no significant differences were observed for pod number. Inoculation increased TGW and pod number by an average of 17 and 31%, respectively, compared with non-inoculated control (Table 5). Irrespective of soybean cultivar, TGW for the tested *Bradyrhizobium* isolates was in the order: USDA110 = GEM96 ≥ GMM36 ≥ GMF14 > no inoculation. However, pod numbers did not differ statistically among the tested strains. Significantly higher TGW was observed in Siroca and Sultana independent of *Bradyrhizobium* inoculation. Inoculation significantly increased TGW in Siroca and Sultana but not in Merlin. The highest TGW of 148 g was reached in Siroca inoculated with USDA110. Soybean cultivars differed in pod number, with Sultana and Merlin showing significantly higher numbers than Siroca.

**TABLE 5:** Effects of different Bradyrhizobium strains inoculated to three soybean cultivars and their interaction on grain yield, pod number, protein yield, and thousand-grain weight under field conditions.

Average values		Pod no.	TGW (g)	Protein content (g kg^–1^)	Oil content (g kg^–1^)	Grain yield (t ha^–1^)
**Cultivar**						
Siroca		14 b	128 a	407 a	216 b	1.27b
Sultana		17 a	130 a	379 b	230 a	1.32a
Merlin		15 ab	107 b	378 b	228 a	1.12c
Mean SED (*p* = 0.05)		1.8	5.9	9.21	7.62	0.04
**Bradyrhizobia**						
No inoculation		12 c	107 c	326 c	254 a	0.99d
GMF14		15 ab	117 b	403 ab	216 bc	1.20c
GMM36		15 ab	125 ab	391 b	225 b	1.25c
GEM96		16 a	128 a	412 a	213 c	1.38ab
USDA110		17 a	131 a	408 a	214 bc	1.40a
Mean SED (*p* = 0.05)		2.8	9.0	13.90	11.51	0.07

**Cultivar**	**Bradyrhizobia**					

Siroca	No inoculation	13	110 de	345	244	0.99fg
	GMF14	14	118 cde	415	209	1.23cde
	GMM36	15	128 bcd	413	213	1.26bcd
	GEM96	16	135 abc	426	208	1.43a
	USDA110	14	148 a	435	206	1.41ab
Sultana	No inoculation	15	110 de	310	263	1.08ef
	GMF14	17	127 bcd	400	221	1.24cd
	GMM36	17	143 ab	385	232	1.45a
	GEM96	16	135 abc	399	220	1.32abc
	USDA110	18	133 abc	403	215	1.47a
Merlin	No inoculation	12	101 e	322	256	0.89g
	GMF14	16	107 e	395	219	1.12def
	GMM36	15	104 e	376	231	1.06f
	GEM96	17	112 de	411	211	1.23cde
	USDA110	19	112 de	386	222	1.33abc
Mean SED (*p* = 0.05)		-	19.5	-	-	0.15

*Values followed by the same letter(s) in each column are not significantly different at p ≤ 0.05. TGW, Thousand-grain weight; SED, Standard Error of a Difference.*

### Grain Yield

Grain yield varied significantly among the soybean cultivars, *Bradyrhizobium* strains, and soybean cultivar × *Bradyrhizobium* interaction (Table 4). Generally, inoculation significantly increased grain yield by an average of 32% compared with the non-inoculated control. A significantly higher grain yield of 1.32 t ha^–1^ was obtained in Sultana, while the lowest was obtained in Merlin (Table 5). Among the tested strains, inoculation with USDA110 and GEM96 significantly increased grain yield up to 1.40 and 1.38 t ha^–1^, respectively, while the lowest yield, 0.99 t ha^–1^ was observed in the non-inoculated control. A significantly higher grain yield, 1.47 t ha^–1^ was observed in Sultana in combination with USDA110 and was statistically similar to Sultana-GMM36, Siroca-GEM96, Siroca-USDA110, Sultana-GEM96, and Merlin-USDA110. The lowest grain yield was observed in non-inoculated Merlin.

### Protein and Oil Content

Protein and oil contents were significantly influenced by soybean cultivar and *Bradyrhizobium* strain inoculation (Table 4). No significant soybean cultivar × *Bradyrhizobium* interactions were detected for the protein and oil contents. Soybean cultivars differed in protein content, with Siroca yielding significantly more protein content (407 g kg^–1^) irrespective of *Bradyrhizobium* inoculation (Table 5). Generally, the protein content in grains increased by an average of 24% compared with the non-inoculated control. Among the tested strains, inoculation with GEM96 resulted in a significantly higher protein content of 412 g kg^–1^ and was statistically similar to USDA110 and GMF14. Sultana and Merlin had the highest significant oil contents, 230 and 228 g kg^–1^, respectively. In contrast to the protein content, the non-inoculated control yielded the highest oil content, 254 g kg^–1^, while the lowest oil content was observed in GEM96.

## Discussion

### Nodulation and N Uptake in Greenhouse and Field Conditions

The soils for both greenhouse experiments were collected from the same site where the field trial was conducted. However, significantly higher nodulation and shoot N uptake were observed in the greenhouse compared with the field conditions, indicating the strong influence of abiotic variables on nodulation and soybean growth (Hungria and Vargas, 2000; Goyal et al., 2021). The observed low mean temperature (11.4°C) in field conditions in early May translated into a low root zone temperature (Schmidt et al., 2015), and low precipitation (5 mm, i.e., only 20% of total precipitation) during germination and seedling establishment of soybean plants likely influenced the symbiotic performance of the strains. This potentially impacted nodulation and resulted in a substantial reduction in biologically derived N (Márquez-García et al., 2015). Thus, early drought and low temperature are important factors in regard to efficient N fixation and enhancement of soybean productivity traits. Other studies have reported induced nodule senescence, reduced leghemoglobin content, and BNF in leguminous plants grown under drought conditions compared with their counterparts in well-watered soils (Ruiz-Lozano et al., 2001; Márquez-García et al., 2015; Kibido et al., 2020). As expected, the moisture regime influenced the shoot biomass increase but did not correspond with N uptake, likely as a result of the dilution effect due to the higher dry matter accumulation in the well-watered conditions (Jarrell and Beverly, 1981).

We observed 2–4 times more nodules in both greenhouse experiments compared with the field trial, which corresponded with over three times higher soybean shoot N uptake. Nodule numbers in both greenhouse experiments were within the range of values reported for sites with a soybean-growing history in Muncheberg and Fehrow, Northeast Germany (Halwani et al., 2021) and Central Germany (Zimmer et al., 2016). These values are, however, lower than those in a report that used metadata involving numerous field trials in several countries and climates (Thilakarathna and Raizada, 2017), re-echoing low soybean-rhizobium symbiosis in the Northeastern agro-climatic environment of Germany.

### Nodulation Response of Strains to Stressed Conditions

Our results showed that the present indigenous Bradyrhizobium strains from Northeast Germany were able to nodulate a range of soybean cultivars adapted to Central European conditions. The significant strain × cultivar interaction in most measured parameters suggests greater specificity, in agreement with Mason et al. (2021), who reported that all indigenous isolates from the Philippines were able to form nodules on almost all soyean cultivars.

The performance of the strains, however, varied based on their combination with specific soybean cultivars under different soil moisture conditions. While inoculation of Merlin, in general, showed low nodulation and biomass yield under normal moisture conditions, there was enhanced nodulation with GEM96 under field conditions with drought stress. The superior effects of GEM96 were minimal when inoculated to Sultana in greenhouse conditions, implying a better symbiotic relationship of Merlin with GEM96 in drought agro-climates. Yuan et al. (2020), in agreement with our results, observed that cold-tolerant GMF14 strain inoculation to Merlin yielded the highest biomass growth under low-temperature conditions. Other reports also observed higher potentials for rhizobium symbiosis, nodulation, and BNF among local cultivars than in breeding lines (Provorov and Tikhonovich, 2003; Kouas et al., 2005; Rodiño et al., 2011). These results imply that the symbiotic performance of Merlin, a cultivar well adapted to the growing conditions in Northeast Germany (Karges et al., 2022), could be enhanced under drought conditions by inoculation with GEM96.

The tolerance of rhizobium strains to drought reflects their ability to remain viable in the soil and is linked to the genetic traits that affect BNF, nodulation, survival, growth rate, and specificity with the host plant (Zaman-Allah et al., 2007). Their efficiency in colonizing roots likely contributes to executing beneficial plant growth-promoting activities (Kibido et al., 2020). On the other hand, the ability of the host plant to enhance BNF under drought conditions is linked to its sustained supply of photosynthates to the nodules (King and Purcell, 2005). Thus, a combination of these traits could be essential to mitigate the effects of drought.

The observed cultivar-by-strain interaction in varying soil moisture conditions reflects the impacts of the environment wherein the strains were isolated and thus presents opportunities to harness cultivar-Bradyrhizobium specificity for enhanced soybean productivity (Gunnabo et al., 2019). According to Giller (2001) and Grossman et al. (2011), the impacts of the environment, management, and soil edaphic features could influence the environmental resiliency of strains to nodulate in extreme conditions. Strains GEM96 and GMM36 were isolated from a drought-prone arable field in Müncheberg, while GMF14 was isolated in Fehrow with a similar agro-climate but under varying management practices. Thus, micro conditions at the field scale likely influenced the adaptive abilities of the strains. This implies that the strains newly introduced by inoculation with commercial inoculants could survive and remain viable in the soil, and their interaction with the immediate environment is likely to enhance stress-induced genetic changes which would be relevant for their adaptation. A report by Zhang et al. (2003), similar to our observations, showed that rhizobium isolates from a northern-adapted climate were more effective under cool conditions. Numerous biotic and abiotic factors, such as N limitation and drought stress, have been postulated to be important factors that affect the symbiotic performance of persisting rhizobium strains previously introduced to the soil by inoculation (Squartini et al., 1993; Bordeleau and Prévost, 1994; Bano et al., 2010; Thilakarathna and Raizada, 2017).

Although nodulation parameters and biomass yield are important traits for assessing host-rhizobium performance (Voisin et al., 2003), we did not analyze other important plant metabolites, such as coumestrol, abscisic acid, reactive oxygen species, and nitrogenase activity, which are produced or altered in response to drought (Wilkinson and Davies, 2009; Tripathi et al., 2016). Future research should include important bioassays that may be necessary to explain the efficiency of this plant-rhizobium interaction.

### Symbiotic Effectiveness of Local Isolates Compared With Standard Strain Under Field Conditions and Their Compatibility With Soybean Cultivars

The present study confirms the importance of inoculation for enhanced soybean yield in Europe (Albareda et al., 2009; Zimmer et al., 2016; Halwani et al., 2021). Altogether, inoculation with our isolates significantly increased the protein content, grain yield, TGW, and pod number up to an average of 24, 32, 17, and 31%, respectively. Zimmer et al. (2016) observed similar protein contents and twice higher grain yield values using commercial inoculants in Central Germany. The grain yield, TGW, and pod number were positively correlated with nodule number and nodule dry weight at the R3 growth stage. This observation confirms many previous reports that enhanced nodulation is essential for increased soybean grain yield (Albareda et al., 2009; Thilakarathna and Raizada, 2017; Moretti et al., 2018).

In the sampled sites, commercial HiStick^®^-soy inoculum containing high N_2_ fixing *B. japonicum* was introduced between 2013 and 2017 (Zimmer et al., 2016; Yuan et al., 2020). Thus, our isolates that show similarity with strains in commercial inoculants may have benefitted from the horizontal transfer of symbiotic genes from commercial inoculants (Zimmer et al., 2016; Yuan et al., 2020). Horizontal gene transfer is an adaptation mechanism in bacteria and may occur within several years after introducing commercial inoculants (Barcellos et al., 2007; Soucy et al., 2015). Strain GEM96 was isolated from a site with no soybean history but was cultivated with uninoculated alfalfa in the past.

Strain GEM96, GMF14, and USDA110 inoculation yielded acceptable protein contents (403–412 g kg^–1^) for human consumption and were within the range reported for early maturity soybean groups in Austria (Vollmann et al., 2000). The highest grain yield was achieved by the Sultana-USDA110, Sultana-GMM36, and Siroca-GEM96 combinations. This significant strain × cultivar interaction suggests the need to match certain cultivars with specific strains to improve soybean yield. Therefore, inoculation of GEM96 to Siroca or Sultana presents an option to increase soybean grain yield in Northeast Germany. The symbiotic relationship of Merlin with GEM96 for biomass production did not translate into increased grain yield, likely as a result of limited rainfall during the reproductive growth stage of soybean plants.

The observed yield in the present study was lower than the average reported by Reckling et al. (2020) in 16 monitored farm trials in northeastern Germany during the 2014–2016 growing year. This is ascribed to the drought conditions, which particularly marred the optimum growth of soybean plants. The annual precipitation in the trial year was 440 mm, and the cumulative rainfall amount during the critical growth of soybean (May–August) was only 164 mm, a level short of 90 mm compared with the average in the last 20 years (Supplementary Table 2). In this regard, even though the present isolates may possess the ability to thrive under drought and cold stress conditions, this trait may only be relevant immediately after sowing or seedling establishment, where an adequate rhizobium population is necessary for successful inoculation (Albareda et al., 2009; Grossman et al., 2011; Chibeba et al., 2018). This indicates that even after successful inoculation, adequate rainfall would be required to obtain optimum crop yield. Thus, further experiments under well-watered conditions are required to evaluate the optimal symbiotic capabilities of the strains. Zimmer et al. (2016) showed that successful soybean cultivation in Europe depends on effective inoculation with non-native *Bradyrhizobium*. Our present study, which agrees with Yuan et al. (2020), demonstrated that there are promising indigenous *Bradyrhizobium* strains that are tolerant to cold temperatures and drought and could potentially be employed to increase soybean BNF and yield in Central Europe. Our results also suggest the need to explore other areas in Europe with longer field-grown inoculated soybean cultivation histories to identify more adapted *Bradyrhizobium* symbionts to expand the pool of inoculum resources in Europe.

## Conclusion

Inoculation of soybean with the present isolates induced higher nodulation, which increased grain yield and protein content compared with the uninoculated control. In particular, the symbiotic performance of the isolates varied based on their combination with specific soybean cultivars under drought and well-watered conditions. There was consistently high nodulation and improved biomass growth in Merlin-GEM96 inoculation in drought conditions but not in well-watered conditions. This, however, did not translate into increased grain yield at harvest. Strain GEM96 inoculation consistently enhanced yield attributes and its combination with Siroca or Sultana resulted in increased grain yield to a similar extent as the standard USDA110 strain. Thus, GEM96 in combination with Siroca or Sultana is effective for grain production under drought-growing conditions in Northeast Germany.

## Data Availability Statement

The original contributions presented in the study are included in the article/Supplementary Material, further inquiries can be directed to the corresponding author/s.

## Author Contributions

RO, KY, KA, and MR conceived and performed the experiments. RO performed all the analyses and wrote the manuscript with assistance from MH, DE, NO-O, and SB-K coordinated the transportation of isolates, and analysis, and provided insightful input into the manuscript write-up. All authors contributed to writing the final version of the manuscript.

## Conflict of Interest

The authors declare that the research was conducted in the absence of any commercial or financial relationships that could be construed as a potential conflict of interest.

## Publisher’s Note

All claims expressed in this article are solely those of the authors and do not necessarily represent those of their affiliated organizations, or those of the publisher, the editors and the reviewers. Any product that may be evaluated in this article, or claim that may be made by its manufacturer, is not guaranteed or endorsed by the publisher.
