# Supplementary material for: Enhanced Soybean Productivity by Inoculation With Indigenous Bradyrhizobium Strains in Agroecological Conditions of Northeast Germany
Source: Front Plant Sci. 2022 Jan 12;12:707080. doi: 10.3389/fpls.2021.707080 (PMC8790476; doi:10.3389/fpls.2021.707080)
Supplement: Supplementary file 1 [file Data_Sheet_1.docx]

Supplementary Material

Table S1: Average monthly temperature in the study area during the 2020 growing season of soybean

| **Parameter** | **Apr** | **May** | **Jun** | **Jul** | **Aug** | **Sep** |
| --- | --- | --- | --- | --- | --- | --- |
| Mean (^⸰^C) | 9.98 | 11.93 | 18.49 | 18.20 | 20.94 | 15.09 |
| SD | 2.79 | 2.85 | 2.51 | 2.19 | 3.11 | 3.17 |
| CV (%) | 28.03 | 23.89 | 13.59 | 12.07 | 14.89 | 21.02 |
| Maximum (^⸰^C) | 16.53 | 17.45 | 23.97 | 23.87 | 27.47 | 21.38 |
| Minimum (^⸰^C) | 2.29 | 5.63 | 12.54 | 12.16 | 14.10 | 8.44 |

SD: standard deviation

CV: coefficient of variation

Table S2: Average monthly precipitation in the study area during the growing season of soybean

| **Year** | **Apr** | **May** | **Jun** | **Jul** | **Aug** | **Sep** |
| --- | --- | --- | --- | --- | --- | --- |
| 1998 | 53.40 | 52.30 | 71.30 | 61.20 | 60.20 | 40.10 |
| 2008 | 91.70 | 4.20 | 23.60 | 39.40 | 66.10 | 54.70 |
| 2013 | 22.90 | 87.60 | 76.90 | 41.40 | 48.60 | 41.20 |
| 2017 | 21.70 | 41.80 | 110.10 | 114.60 | 55.60 | 24.00 |
| 2018 | 27.10 | 15.90 | 26.60 | 97.30 | 17.00 | 20.20 |
| 2019 | 9.60 | 34.50 | 46.00 | 113.20 | 20.50 | 54.40 |
| 2020 | 18.40 | 25.40 | 64.70 | 33.80 | 38.90 | 55.50 |
| Mean (mm) | 34.97 | 37.39 | 59.89 | 71.56 | 43.84 | 41.44 |
| SD | 28.46 | 27.32 | 30.50 | 35.88 | 19.21 | 14.71 |
| CV (%) | 81.38 | 73.08 | 50.93 | 50.15 | 43.81 | 35.50 |
| Maximum (mm) | 91.70 | 87.60 | 110.10 | 114.60 | 66.10 | 55.50 |
| Minimum (mm) | 9.60 | 4.20 | 23.60 | 33.80 | 17.00 | 14.71 |

Table S3: Effects of Bradyrhizobium strains and moisture regimes on nodulation, crop growth and nitrogen uptake in the second greenhouse experiment

| Average values | Shoot dry wt. (g) | N uptake (g kg^-1^) | Nodule no. | Nodule dry wt. (g) |
| --- | --- | --- | --- | --- |
| **Bradyrhizobia (B)** |  |  |  |  |
| No inoculation | 2.15 a | 19.98 b | 4.25 c | 0.02 c |
| GMF14 | 2.29 a | 33.98 a | 37.00 a | 0.14 ab |
| GMM36 | 2.61 a | 33.24 a | 31.50 ab | 0.16 a |
| GEM96 | 2.74 a | 31.90 a | 24.25 b | 0.08 bc |
| USDA110 | 2.45 a | 35.05 a | 35.75 a | 0.13 ab |
| **Moisture (M)** |  |  |  |  |
| Drought | 1.81 b | 31.73 a | 13.80 b | 0.071 b |
| Well-watered | 3.09 a | 29.93 a | 39.30 a | 0.158 a |

Values followed by the same letter(s) on each bar are not significantly different at *p* ≤ 0.05

Table S4: Effects of Bradyrhizobium strains and soybean cultivar on nodulation, crop growth and yield parameters in field conditions

| Average values | Shoot biomass  (t ha^−1^) | Shoot N  (g kg^-1^) | Nodule no. | Nodule dry wt. (g) | LAI | Root biomass  (g plant^-1^) |
| --- | --- | --- | --- | --- | --- | --- |
| **Cultivar (C)** |  |  |  |  |  |  |
| Siroca | 3.32 b | 20.62 a | 12.11 a | 0.60 a | 3.65 a | 0.63 b |
| Sultana | 3.43 b | 21.00 a | 11.37 a | 0.51 b | 2.85 b | 0.95 a |
| Merlin | 3.92 a | 18.27 b | 11.15 a | 0.47 b | 3.23 ab | 0.47 c |
| **Bradyrhizobia (B)** |  |  |  |  |  |  |
| No inoculation | 3.49 a | 15.22 d | 0.6 c | 0.001 c | 2.53 b | 0.58 a |
| GMF14 | 3.54 a | 20.22 c | 13.54 b | 0.05 b | 3.21 ab | 0.66 a |
| GMM36 | 3.68 a | 20.60 bc | 12.33 b | 0.06 b | 3.37 a | 0.72 a |
| GEM96 | 3.63 a | 21.89 ab | 18.20 a | 0.08 a | 3.46 a | 0.68 a |
| USDA110 | 3.41 a | 21.91 a | 13.02 b | 0.06 b | 3.65 a | 0.78 a |

Values followed by the same letter(s) on each bar are not significantly different at *p* ≤ 0.05
